# Diagnostic and Prognostic Value of Serum Omentin-1 in Sepsis: A Prospective Study in Critically Ill Patients

**DOI:** 10.3390/medicina59050833

**Published:** 2023-04-24

**Authors:** Irene Karampela, Natalia G. Vallianou, Dimitrios Tsilingiris, Gerasimos Socrates Christodoulatos, Georgios Antonakos, Ioanna Marinou, Evaggelos Vogiatzakis, Apostolos Armaganidis, Maria Dalamaga

**Affiliations:** 1 Second Department of Critical Care, Attikon General University Hospital, Medical School, National and Kapodistrian University of Athens, 12462 Athens, Greece; 2 Department of Biological Chemistry, Medical School, National and Kapodistrian University of Athens, 11527 Athens, Greece; 3 First Department of Internal Medicine, Evangelismos General Hospital, 10676 Athens, Greece; 4 First Department of Internal Medicine, University Hospital of Alexandroupolis, Democritus University of Thrace, 68100 Alexandroupolis, Greece; 5 Laboratory of Clinical Biochemistry, Attikon General University Hospital, Medical School, National and Kapodistrian University of Athens, 12462 Athens, Greece; 6 Laboratory of Microbiology, Sotiria Athens General Hospital, 11527 Athens, Greece

**Keywords:** adipokine, adipose tissue, biomarker, critically ill, intelectin-1, mortality, omentin-1, sepsis, septic shock

## Abstract

*Background and Objectives*: Omentin-1, also known as intelectin-1, is a novel adipokine with anti-inflammatory activities implicated in inflammatory diseases and sepsis. We aimed to explore serum omentin-1 and its kinetics in critically ill patients early in sepsis and its association with severity and prognosis. *Materials and Methods*: Serum omentin-1 was determined in 102 critically ill patients with sepsis during the first 48 h from sepsis onset and 1 week later, and in 102 age- and gender-matched healthy controls. The outcome of sepsis at 28 days after enrollment was recorded. *Results*: Serum omentin-1 at enrollment was significantly higher in patients compared to controls (763.3 ± 249.3 vs. 451.7 ± 122.3 μg/L, *p* < 0.001) and it further increased 1 week after (950.6 ± 215.5 vs. 763.3 ± 249.3 μg/L, *p* < 0.001). Patients with septic shock (*n* = 42) had higher omentin-1 compared to those with sepsis (*n* = 60) at enrollment (877.9 ± 241.2 vs. 683.1 ± 223.7 μg/L, *p* < 0.001) and 1 week after (1020.4 ± 224.7 vs. 901.7 ± 196.3 μg/L, *p* = 0.007). Furthermore, nonsurvivors (*n* = 30) had higher omentin-1 at sepsis onset (952.1 ± 248.2 vs. 684.6 ± 204.7 μg/L, *p* < 0.001) and 1 week after (1051.8 ± 242 vs. 908.4 ± 189.8 μg/L, *p* < 0.01). Patients with sepsis and survivors presented higher kinetics than those with septic shock and nonsurvivors (Δ(omentin-1)% 39.8 ± 35.9% vs. 20.2 ± 23.3%, *p* = 0.01, and 39.4 ± 34.3% vs. 13.3 ± 18.1%, *p* < 0.001, respectively). Higher omentin-1 at sepsis onset and 1 week after was an independent predictor of 28-day mortality (HR 2.26, 95% C.I. 1.21–4.19, *p* = 0.01 and HR: 2.15, 95% C.I. 1.43–3.22, *p* < 0.001, respectively). Finally, omentin-1 was significantly correlated with the severity scores, the white blood cells, coagulation biomarkers, and CRP, but not procalcitonin and other inflammatory biomarkers. *Conclusions*: Serum omentin-1 is increased in sepsis, while higher levels and lower kinetics during the first week of sepsis are associated with the severity and 28-day mortality of sepsis. Omentin-1 may be a promising biomarker of sepsis. However, more studies are needed to explore its role in sepsis.

## 1. Introduction

Omentin-1 is a recently described adipokine, which is also referred to as intelectin-1 or intestinal lactoferrin receptor [1,2]. Omentin-1 is mainly expressed in visceral adipose tissue, but also in the endothelium, plasma, mesothelial cells, airway goblet cells, the ovaries, and the intestine [3]. It is a 35 kDa hydrophilic polypeptide of 313 amino acids exerting cytokine-like actions. Omentin-1 exhibits insulin-sensitizing, anti-atherosclerotic, and cardiovascular protective effects through the activation of AMP-activated protein kinase (AMPK), which inhibits the nuclear factor κB (NF-κB) and suppresses inflammatory response [3]. Through these mechanisms, omentin-1 is implicated in the regulation of energy homeostasis and immune responses. Indeed, clinical studies have demonstrated that circulating omentin-1 levels are decreased in obesity, metabolic syndrome, type 2 diabetes, cardiovascular diseases, cancer, and chronic inflammation [4,5,6,7,8,9,10,11,12,13,14,15,16,17,18].

Experimental studies have demonstrated that omentin-1 inhibits the NF-κB signaling pathway and the tumor necrosis factor alpha (TNF-α)-induced expression of adhesion molecules, namely vascular cell adhesion molecule 1 (VCAM-1) and intercellular adhesion molecule 1 (ICAM-1), and, thus, blocks the pro-inflammatory actions of TNF-α [19]. Additionally, omentin-1 inhibits lipopolysaccharide (LPS)-induced expression of inflammatory mediators in macrophages [20]. It is noteworthy that omentin-1 is a ligand to lactoferrin and to bacterial-specific carbohydrate residues, and, thus, plays a part in microbial surveillance and recognition [21,22].

Interestingly, other well-known adipokines, such as adiponectin, leptin, resistin, visfatin, chemerin, and the hepatokine fetuin-A, exert immunomodulatory actions (anti-inflammatory, pro-inflammatory, or regulatory) and have been implicated in inflammatory diseases and sepsis [23,24,25,26,27,28,29,30,31,32,33,34]. Our group has previously demonstrated that these adipokines are altered in critically ill patients, with sepsis being associated with the severity and prognosis of sepsis [35,36,37,38,39,40,41]. However, omentin-1 has not been thoroughly studied in sepsis. Recently, a clinical study of critically ill patients showed that increased serum omentin-1 was associated with poor long-term outcomes [42].

Given the anti-inflammatory actions of omentin-1, we hypothesized that serum omentin-1 is altered in sepsis. In this study, we aim to evaluate serum omentin-1 in critically ill patients with sepsis at sepsis onset compared to healthy controls. Moreover, our goal is to investigate serum omentin-1 kinetics during the first week of sepsis and to explore its association with clinical and inflammatory biomarkers as well as with the severity and outcome of sepsis.

## 2. Materials and Methods

### 2.1. Study Design and Participants

We prospectively enrolled consecutive critically ill patients with new onset sepsis hospitalized at the mixed (medical and surgical) adult intensive care unit (ICU) of a tertiary teaching hospital during a 2-year period (August 2013 to July 2015). The inclusion criteria were as follows: (1) adult patients (aged ≥ 18 years); (2) diagnosis of sepsis during the last 48 h. The exclusion criteria were as follows: (1) age <18 years; (2) pregnancy; (3) diabetes mellitus; (4) thyroid disease; (5) liver disease; (6) total parenteral nutrition; (7) malignancy; (8) immunosuppression. We also excluded patients who were hospitalized in the ICU for less than a week from enrollment to the study. We recorded demographic, clinical, and routine laboratory data upon enrollment and 1 week after. We followed patients for 28 days from enrollment to the study and the outcome of sepsis was recorded. The study population was enrolled using the previously established diagnostic criteria of sepsis, and septic patients were initially categorized into three groups: sepsis, severe sepsis, and septic shock [43]. However, the analysis of data was conducted after the new consensus for the definitions of sepsis and septic shock (SEPSIS-3) was reached [44]. In order to update our analysis, we retrospectively recategorized our cases according to the current definitions into two groups: sepsis and septic shock. After applying the new criteria for sepsis, no patient was excluded from the study. We initially enrolled 167 patients. Of these, 65 patients were excluded according to the exclusion criteria, and 102 patients (57 males, aged 64.7 ± 15.6 years) were included in the analysis.

We also enrolled a control group consisting of healthy subjects who were recruited from the outpatient laboratory department. For every patient enrolled, we recruited a healthy subject matched for age (±5 years) and gender. The same exclusion criteria were applied to the control group. We also excluded subjects with clinical signs or history of an acute or chronic infection or inflammatory disease. A total of 102 healthy subjects were included (57 males, aged 66.4 ± 10.3 years). All subjects or their next of kin gave their informed consent for inclusion before they participated in the study. The study was conducted in accordance with the Declaration of Helsinki and its amendments, and the protocol was approved by the Scientific and Ethics Committee of the hospital (#587/10 April 2013).

### 2.2. Laboratory Analysis

We collected blood samples (20 mL) from patients and healthy controls upon enrollment. We also collected blood samples (20 mL) 1 week after enrollment from patients only. Whole blood specimens were immediately centrifuged, and serum was stored at −80 °C. Omentin-1 was determined in serum using a sandwich enzyme immunoassay ELISA kit by Biovendor (#RD191100200R, Brno, Czech Republic). The detection limit was 0.5 μg/L, the inter-assay and intra-assay coefficients of variations were between 4.4–4.8% and 3.2–4.1%, respectively, while the linear range of the assay was within 50–644 μg/L. We also measured inflammatory biomarkers (C-reactive protein and procalcitonin), interleukins (IL) 1β, 6 and 10, and soluble urokinase-type plasminogen activator receptor (suPAR) as previously described [35,38,40,41]. Homeostasis model assessment score of insulin resistance (HOMA-IR) was calculated using the following formula: [fasting serum insulin (μU/mL) × fasting serum glucose (mmol/L)]/22.5.

### 2.3. Statistical Analysis

The statistical analysis was performed using the statistical package IBM-SPSS^®^ Statistics for Windows, Version 24.0. Armonk, NY, USA: IBM Corp. Categorical variables were assessed using the chi-square test. The Shapiro–Wilk test was employed to examine the normality hypothesis. Normally distributed variables were analyzed using the *t*-test and paired *t*-test, while for non-normally distributed variables we used the Mann–Whitney U and Wilcoxon matched-pair tests. For continuous variables, we used the Spearman correlation coefficients (r) as a measurement of correlation. For survival analysis, we used the Kaplan–Meier method, and we generated the survival curves, while the log rank test was used for comparisons. The receiver operating characteristic (ROC) curves were assessed to calculate the discriminating power of selected biomarkers to distinguish sepsis from septic shock. The comparison of ROC curves was performed using the DeLong test in the MedCalc^®^ statistical software version 20.218 (MedCalc Software Ltd., Ostend, Belgium). We also performed a multivariate Cox-regression analysis, adjusting for acute physiology and chronic health evaluation II score (APACHE II) and statistically significant laboratory biomarkers of sepsis in order to determine the independent predictors of 28-day mortality among statistically significant inflammatory biomarkers. A two-sided *p* value of less than 0.05 was considered significant.

## 3. Results

### 3.1. Baseline Characteristics of Patients and Controls

The baseline demographic, clinical, and laboratory data of patients and controls are depicted in Table 1. According to the SEPSIS-3 criteria, 42 patients had septic shock at enrollment and 60 had sepsis. Pulmonary and abdominal sepsis were most common (35% and 24% respectively), while the causative pathogen was identified in 60 cases as follows: gram-negative bacteria in 36 cases (60%); gram-positive bacteria in 14 cases (23%); and fungi in 10 cases (17%). During the 28-day period of follow up, 30 patients died, namely 6 of the 60 patients with sepsis (mortality rate 10%) and 24 of the 42 patients with septic shock (mortality rate 57%). The patients and controls were age and gender-matched as per the protocol. Furthermore, the BMI did not differ significantly between the two groups (Table 1). The hematologic, coagulation, and main metabolic indices as well as the C-reactive protein (CRP) were significantly different between patients and controls (Table 1).

### 3.2. Serum Omentin-1 in Patients and Controls

**Figure 1:**
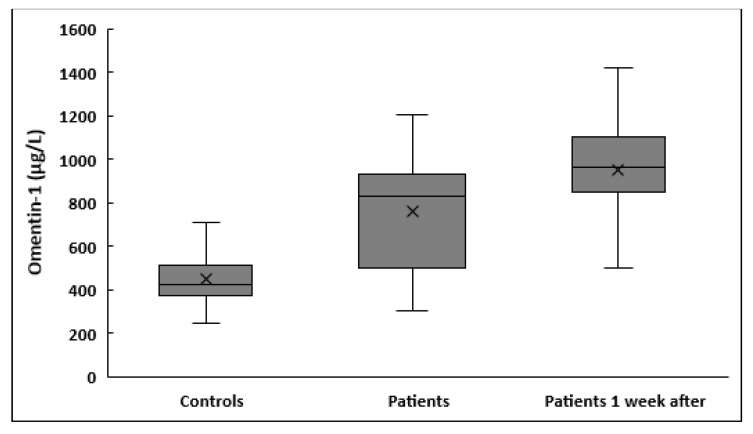
Box plots of serum omentin-1 in healthy controls and in patients with sepsis at enrollment and 1 week after.

Serum omentin-1 at enrollment was significantly higher in patients compared to controls (763.3 ± 249.3 vs. 451.7 ± 122.3 μg/L, *p* < 0.001) (Table 1). In critically ill patients with sepsis, serum omentin-1 increased significantly 1 week after enrollment to the study (950.6 ± 215.5 vs. 763.3 ± 249.3 μg/L, *p* < 0.001) (Figure 1).

### 3.3. Serum Omentin-1 and Sepsis Severity

**Figure 2:**
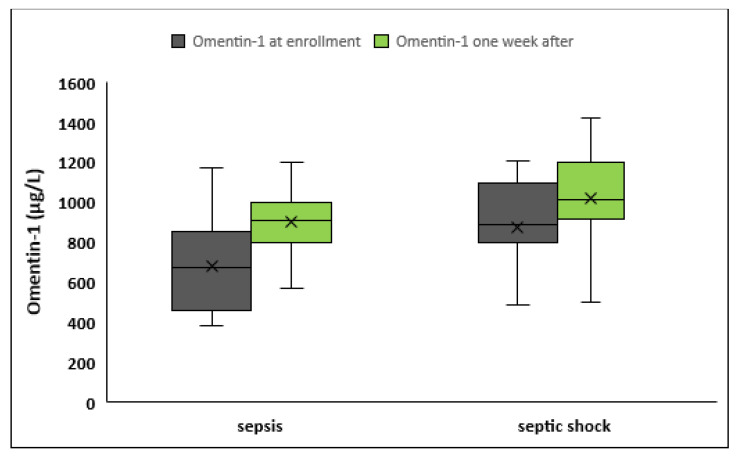
Serum omentin-1 in patients with sepsis (*n* = 60) and in patients with septic shock (*n* = 42) at enrollment and 1 week after.

Patients who presented with septic shock at enrollment (*n* = 42) had significantly higher omentin-1 than those who presented with sepsis (*n* = 60) both at sepsis onset (877.9 ± 241.2 vs. 683.1 ± 223.7 μg/L, *p* < 0.001) and 1 week after (1020.4 ± 224.7 vs. 901.7 ± 196.3 μg/L, *p* = 0.007) (Table 2, Figure 2). Regarding kinetics of omentin-1 during the first week of sepsis, omentin-1 increased significantly in both groups. However, patients with sepsis exhibited a significantly higher absolute as well as higher percentage change from baseline during the first week of sepsis compared to patients with septic shock (Δ(omentin-1): 218.6 ± 145.6 vs. 142.5 ± 143.5, *p* = 0.01; Δ(omentin-1)% 39.8 ± 35.9% vs. 20.2 ± 23.3%, *p* = 0.01, respectively).

**Figure 3:**
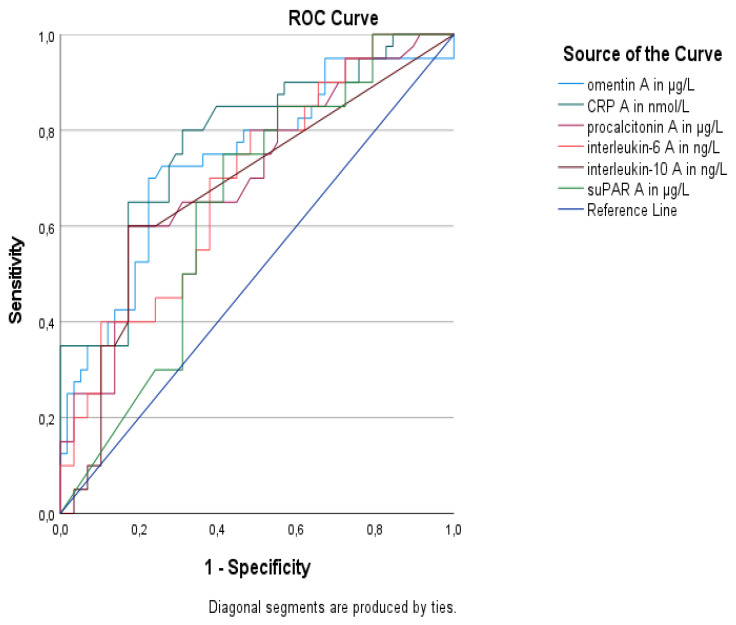
Area under the receiver operating characteristic curve (AUROC) distinguishing sepsis from septic shock in 102 patients with sepsis. Decimal separators are denoted by commas instead of dots.

The ROC analysis of serum omentin-1 and other inflammatory biomarkers for the discrimination between sepsis and septic shock at enrollment is shown in Table 3. Serum omentin-1 at enrollment (AUROC > 0.739) performed similarly with CRP (AUROC > 0.778), procalcitonin (AUROC > 0.707), IL-6 (AUROC > 0.69), IL-10 (AUROC > 0.678) and suPAR (AUROC > 0.64) in distinguishing sepsis from septic shock, as the comparison of the ROC curves did not yield any statistically significant results (*p* > 0.05 derived from the DeLong test) (Figure 3).

**Figure 4:**
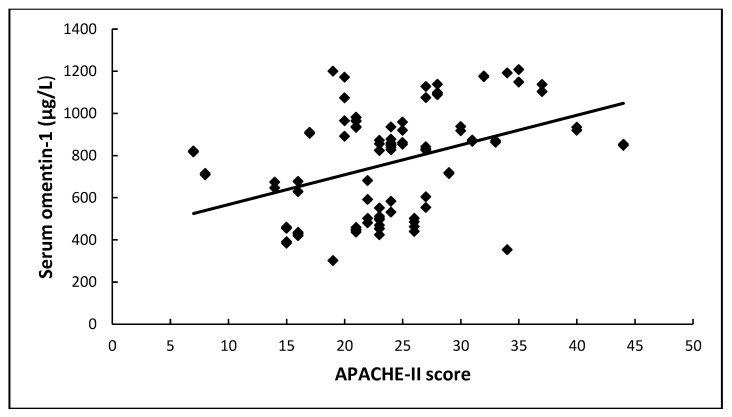

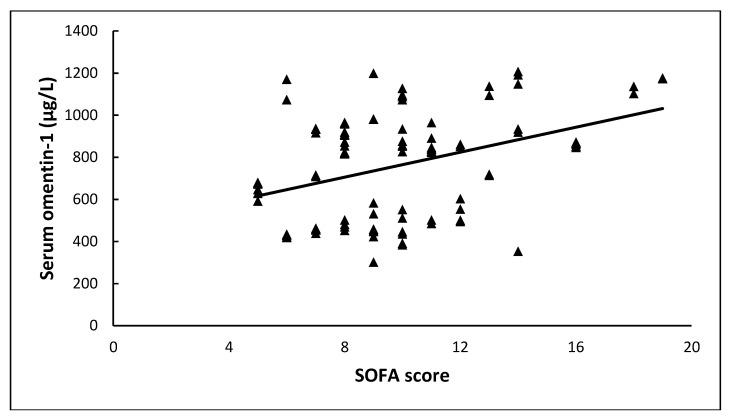
Serum omentin-1 is significantly associated with APACHE II and SOFA scores at sepsis onset in 102 critically ill patients.

Finally, omentin-1 at enrollment presented a significant positive association with the severity scores APACHE II and SOFA (r = 0.44, *p* < 0.001 and r = 0.34, *p* < 0.001, respectively) (Figure 4).

### 3.4. Serum Omentin-1 and Sepsis Outcome

**Figure 5:**
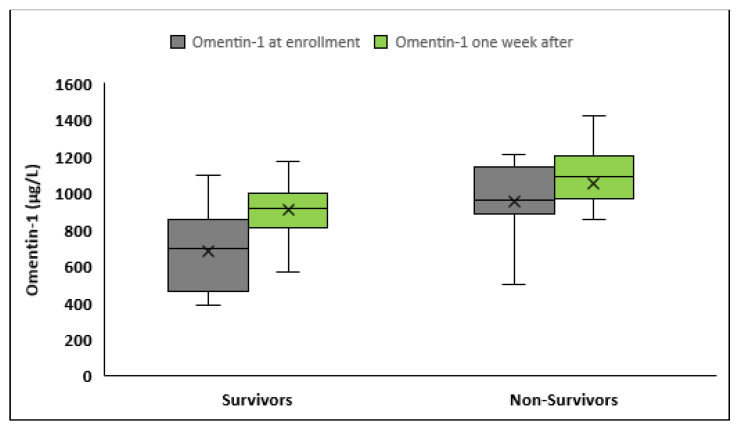
Box plots of serum omentin-1 at enrollment and 1 week after according to the outcome of sepsis at 28 days after sepsis onset.

During the 28 days of follow up from enrollment to the study, 30 patients died and 72 survived sepsis. Serum omentin-1 was significantly higher in nonsurvivors compared to survivors both at enrollment (952.1 ± 248.2 vs. 684.6 ± 204.7 μg/L, *p* < 0.001) and 1 week after (1051.8 ± 242 vs. 908.4 ± 189.8 μg/L, *p* < 0.01) (Figure 5). While omentin-1 increased significantly 1 week after sepsis onset in both groups (*p* < 0.001), survivors had a greater mean increase (Δ(omentin-1): 223.8 ± 147.9 μg/L vs. 99.6 ± 111.3 μg/L, *p* < 0.001), and percentage change from baseline (Δ(omentin-1)% 39.4 ± 34.3% vs. 13.3 ± 18.1%, *p* < 0.001) compared to nonsurvivors.

**Figure 6:**
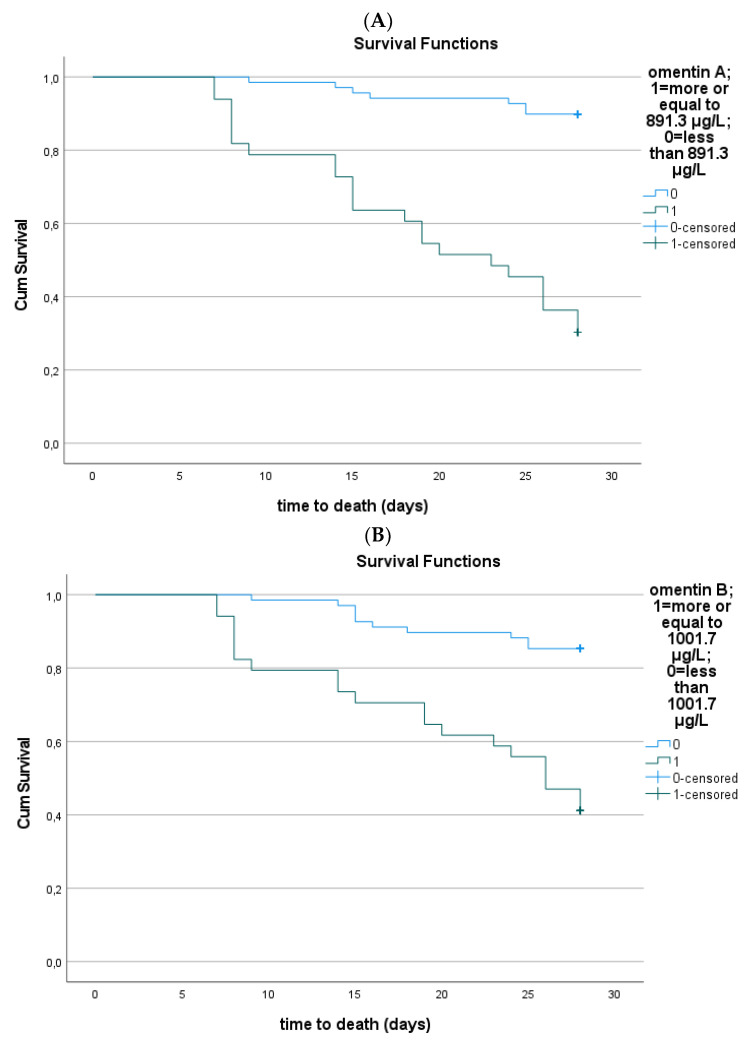

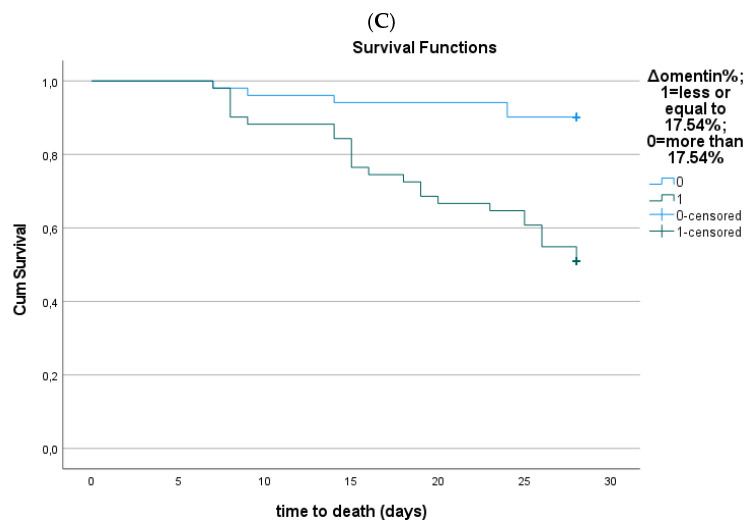
(**A**) Kaplan–Meier estimates of mortality in 102 septic patients based on serum omentin-1 at enrollment cutoff values obtained via ROC analysis (log rank test: 45.1, *p* < 0.001). (**B**) Kaplan–Meier estimates of mortality in 102 septic patients based on serum omentin-1 1 week after enrollment cutoff values obtained via ROC analysis (log rank test: 23.5, *p* < 0.001). (**C**) Kaplan–Meier estimates of mortality in 102 septic patients based on percentage change in serum omentin-1 from baseline cutoff values obtained via ROC analysis (log rank test: 18.27, *p* < 0.001). Decimal separators are denoted by commas instead of dots, in the y axis.

The Kaplan–Meier survival curves showed that patients with lower omentin-1 at sepsis onset and 1 week after had improved 28-day survival (*p* < 0.001) (Figure 6A,B). The cutoff value of omentin-1 was estimated at 891.3 μg/L at enrollment and 1001.7 μg/L 1 week after enrollment. Furthermore, patients presenting a higher percentage change in serum omentin-1 from baseline also had improved 28-day survival (*p* < 0.001), with an estimated cutoff value of 17.54%.

Unadjusted Cox regression analyses demonstrated that serum omentin-1 at enrollment (HR: 1.005, 95% C.I. 1.003–1.007, *p* < 0.001) and 1 week after (HR:1.003, 95% C.I. 1.001–1.005, *p* < 0.001) was significantly associated with 28-day mortality of sepsis. After adjustment for the APACHE II score and statistically significant laboratory biomarkers of sepsis, higher omentin-1 at enrollment was independently associated with 28-day mortality (HR 2.26, 95% C.I. 1.21–4.19, *p* = 0.01). One week after sepsis onset, higher omentin-1 was also an independent predictor of 28-day mortality (HR 2.15, 95% C.I. 1.43–3.22, *p* < 0.001) (Table 4). Noteworthily, IL-6 1 week after sepsis onset, but not CRP, was also independently associated with mortality.

### 3.5. Correlations of Serum Omentin-1 and Other Biomarkers

Spearman correlations between serum omentin-1 and laboratory biomarkers of sepsis are depicted in Table 5. Concerning the hematologic biomarkers, omentin-1 exhibited significant positive correlation only with the white blood cells at enrollment and 1 week after. Furthermore, neutrophils 1 week after sepsis onset were positively associated with omentin-1. All coagulation biomarkers were significantly correlated with omentin-1 at enrollment. Regarding metabolic biomarkers, baseline lactate presented a positive correlation of marginal significance, while baseline creatinine was significantly correlated with omentin-1. Although glucose and insulin at enrollment were not correlated with omentin-1, HOMA-IR presented a significant positive correlation. Notably, omentin-1 did not correlate with BMI in septic patients. Finally, omentin-1 at sepsis onset presented a significant positive correlation only with CRP, but not with procalcitonin, IL-1β, IL-6, IL-10, or suPAR.

## 4. Discussion

In this prospective observational study, we investigated serum omentin-1 in critically ill patients with new onset sepsis during the first week from enrollment. We found that serum omentin-1 was significantly increased compared to healthy controls and increased further 1 week after enrollment in all patients. Moreover, omentin-1 was higher in patients presenting with septic shock, the more severe presentation of sepsis, but exhibited lower kinetics compared to those presenting with sepsis. Regarding prognosis, we demonstrated that omentin-1 was lower during the first week of sepsis but presented a higher absolute and percentage change (increase) in patients who survived 28 days from sepsis onset compared to nonsurvivors. Finally, we showed that higher serum omentin-1 at sepsis onset as well as 1 week later was an independent predictor of 28-day mortality in critically ill patients with sepsis.

Omentin-1 is a newly discovered adipokine, which exerts cytokine-like actions. Experimental evidence has highlighted an anti-inflammatory role of omentin-1 [3,45,46]. In particular, omentin-1 has been shown to inhibit the TNF-α-induced cyclooxygenase (COX)-2 expression in vascular endothelial cells through blocking of the c-Jun N-terminal kinase (JNK) activation possibly by activation of the AMPK pathway [47]. Another experimental study has demonstrated that omentin-1 prevents the TNF-α-induced expression of the adhesion molecule VCAM-1 in smooth muscle cells through inhibition of the activation of p38 and JNK [48]. Moreover, omentin-1 was found to inhibit TNF-α-activated NF-κB by preventing NF-κB inhibitory protein (IκBα) degradation and NF-κB/DNA binding activity and to block TNF-α-induced expression of adhesion molecules VCAM-1 and ICAM-1 in human umbilical vein endothelial cells [49]. These experimental data suggest that omentin-1 acts as an anti-inflammatory molecule by blocking the TNF-α dependent inflammatory responses [19,45]. Furthermore, an experimental study has demonstrated that omentin-1 also inhibits LPS-induced expression of pro-inflammatory cytokines in macrophages [20]. In agreement with this finding, animal studies have demonstrated that omentin-1 may protect against LPS and bleomycin-induced acute lung injury [50,51].

Clinical studies have highlighted that omentin-1 is decreased in obesity and related metabolic diseases, with low levels reflecting cardiometabolic risk [5,6,7,8,9,13,52,53,54]. Thus, omentin-1 has been proposed to be the missing link between obesity and cardiovascular disease exerting protective effects against metabolic syndrome, diabetes, and related cardiovascular diseases through attenuation of low-grade chronic inflammation [4,13]. An experimental study demonstrated that omentin-1 inhibits resistin-induced expression of toll-like receptor 4 (TLR4) as well as NF-κB phosphorylation in cardiomyoblasts, thus, preventing resistin-induced cardiac inflammation and hypertrophy [55].

Although omentin-1 has been mostly studied as a biomarker of obesity and the metabolic syndrome, related to insulin resistance and diabetes, it has also drawn attention for its role in inflammatory diseases. In an animal model of ulcerative colitis, omentin-1 was shown to attenuate inflammation by inhibiting the expression of endoplasmic reticulum stress-related proteins [56]. Noteworthily, in a clinical study of 192 patients with inflammatory bowel disease (IBD), serum omentin-1 was significantly decreased, and low levels independently predicted disease activity [18]. In contrast to the findings observed in IBD, omentin-1 was significantly increased in 40 patients with juvenile idiopathic arthritis compared to 26 healthy controls being associated with the presence of arthritis and the number of joints involved [57]. Additionally, plasma omentin-1 was found to be increased in patients with systemic lupus erythematosus and psoriasis who presented with arthritis but not in those without arthritis [17]. However, in a clinical study, patients with rheumatoid arthritis had lower levels of omentin-1 in the synovial fluid compared to patients with osteoarthritis [58].

Omentin-1 has been linked to the pathogenesis of atopic inflammation. In a recent animal study, expression of omentin-1 was significantly increased in asthmatic airways and in skin lesions of atopic dermatitis [59]. In line with this finding, omentin-1 has been recognized as a prominent component of pathologic mucus in patients with acute severe asthma in conjunction with eosinophilic airway inflammation [60]. Noteworthily, evidence supports a host defense role of omentin-1 against pathogenic bacteria in the airways as well as in the intestine due to its ability to bind lactoferrin, recognize bacteria-specific structures, and enhance phagocytic clearance of pathogenic microorganisms [21,61,62,63]. However, the role of omentin-1 has not been investigated in viral infectious diseases. In a recent clinical study, serum omentin was significantly decreased in patients with coronavirus disease 2019 (COVID-19) compared to healthy controls [64]. Therefore, further studies are needed to determine the role of omentin-1 in acute and chronic inflammatory diseases.

Omentin-1 has not been thoroughly studied in sepsis. There are only two previous studies on circulating omentin-1 in critically ill patients with or without sepsis [42,65]. In agreement with our findings, the study by Luedde et al. showed that higher omentin-1 at admission was an independent predictor of long-term mortality (median follow up of 353 days, range 29−800 days) [42]. However, this study did not demonstrate any significant difference between serum omentin-1 in 117 critically ill patients and 50 healthy controls, nor between 84 critically ill patients with sepsis and 33 critically ill patients without sepsis. This may be explained by the differences in the study population. The healthy subjects in the study by Luedde et al. were younger than patients and their BMI was not reported, while in our study the cases and controls were age- and gender-matched and the BMI was not significantly different [42]. Since serum omentin-1 has been shown to be inversely related to BMI, this may have been a confounding factor in the study by Luedde et al. [5,42,66,67]. Moreover, our patients had more severe diseases, as reflected by the higher APACHE II scores. Of note, we studied only critically ill patients with new onset sepsis (within 48 h), while Luedde et al. did not report the timing of sepsis onset in their cases [42]. As we have shown, serum omentin-1 significantly increases 1 week after sepsis onset in all patients. Therefore, the timing of omentin-1 measurement in relation to sepsis onset may be an important factor explaining the differences in the results. Finally, Luedde et al. used a different ELISA kit for the determination of omentin-1 without reporting the characteristics of this kit (coefficients of variation and detection limit) and the actual omentin-1 values [42]. Hence, we cannot directly compare our findings.

The study by Gültekin et al. investigated serum omentin-1 in 154 surgical critically ill patients measured at multiple time points (upon admission, upon changes in vital signs, clinical care and oral nutrition, and at sepsis onset and at discharge) [65]. The authors reported a total of 423 omentin-1 measurements. In contrast to our results, they found that omentin-1 was higher in patients without sepsis and in those who were discharged from the ICU compared to septic patients and those who died in the ICU [65]. However, the study population was substantially different from our patients. These were only surgical patients, younger than our study population, with less severe disease and a better prognosis, as reflected by the lower APACHE II and SOFA scores and the lower ICU mortality. Additionally, this study reported ICU mortality, in contrast to our study that reports 28-day mortality, while the length of ICU stay was not reported. Noteworthily, the authors did not define the time point of reported omentin-1 values. One may assume that they analyzed all measurements during the ICU stay in all patients, regardless of the timing and the relation to the clinical course or sepsis onset.

**Figure 7:**
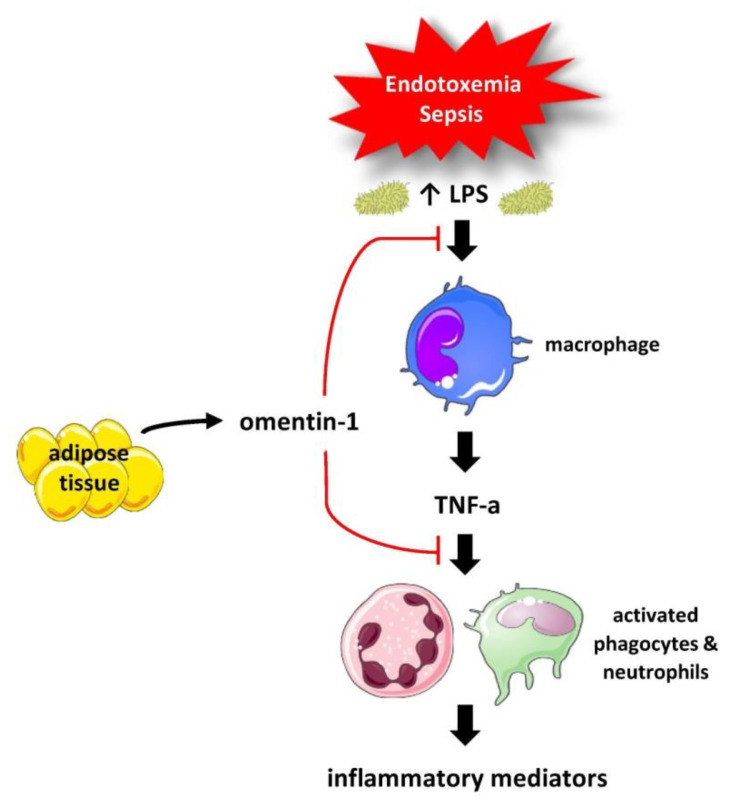
Omentin-1 interferes with important inflammatory signals, preventing key factors from propagating inflammation. Abbreviations: LPS, lipopolysaccharide; TNF-a, tumor necrosis factor alpha (images are originated from the free medical site http://smart.servier.com/ by Servier licensed under a Creative Commons Attribution 3.0 Unported License). https://smart.servier.com/ (accessed on 15 March 2023).

The systemic inflammatory response characterized by the activation of the innate immunity by pathogens, the initiation of multiple signaling pathways leading to the secretion of pro-inflammatory mediators, and the outset of the cytokine storm comprise key mechanisms in the pathophysiology of sepsis [68]. Omentin-1 interferes with important inflammatory signals, preventing key factors, such as LPS and TNF-α, from propagating inflammation (Figure 7). We hypothesized that omentin-1 is altered in sepsis. Our study showed that higher serum omentin-1 is associated with the severity of sepsis, presenting significant correlations with the severity scores. Moreover, our study demonstrated that increased circulating omentin-1 levels early in sepsis predict a poor outcome, despite its anti-inflammatory activity. This finding may be explained by a compensatory role of omentin-1 in sepsis, i.e., increased omentin-1 may reflect an augmented inflammatory response, the severity of sepsis, and a poor outcome. Nonetheless, the survival analysis revealed that lower baseline values and a higher percentage increase in omentin-1 during the first week from sepsis onset was associated with increased survival. This finding complies with the anti-inflammatory actions of omentin-1.

In this study, we comparatively investigated the performance of various inflammatory biomarkers at sepsis onset in discriminating sepsis from septic shock. We found that the discriminative ability of serum omentin-1 to distinguish sepsis from septic shock was similar to CRP and procalcitonin. We further explored the association of serum omentin-1 with inflammatory, coagulation, and metabolic parameters. We found that omentin-1 is positively associated only with white blood cells, neutrophils, and CRP but not procalcitonin, IL-1β, IL-6, IL-10, and suPAR. One possible explanation is that inflammatory biomarkers as well as omentin-1 are not specific to sepsis. However, the coagulation biomarkers at baseline exhibited significant positive correlations with omentin-1, reflecting the implication of coagulation in sepsis. Lactate, which has a well-known predictive value in sepsis, showed a positive association of borderline significance with omentin-1. Baseline creatinine as well as HOMA-IR exhibited positive associations, reflecting metabolic derangement in sepsis. Of note, we did not find any significant association with BMI, possibly because omentin-1 alterations in the acute phase of sepsis surpass the effect of BMI.

Adiponectin is a classic adipokine exerting similar metabolic and anti-inflammatory actions to those of omentin-1 [12,69]. These two adipokines promote insulin sensitivity and glucose tolerance by inhibiting TNF-α and LPS inflammatory signals [23]. The alterations and kinetics of serum adiponectin in critically ill patients with sepsis have been previously investigated by our group [35,36]. Interestingly, we found that both serum omentin-1 and adiponectin increased in critically ill patients with sepsis compared to controls and were higher in patients with septic shock compared to those with sepsis at enrollment and 1 week after. We also showed that they were both higher in nonsurvivors than survivors at 28 days. Regarding kinetics, higher kinetics of both omentin-1 and adiponectin were associated with improved 28-day survival. Finally, adiponectin—similar to omentin-1—was significantly associated with severity scores and CRP. Our results indicate that adiponectin and omentin-1 may share common pathophysiologic roles in patients with sepsis.

This is the first study to explore omentin-1 and its kinetics during the first week from sepsis onset as well as its association with sepsis severity and outcome. The prospective case-control design including matching of age and gender in cases and controls, the careful selection of cases according to specific inclusion and exclusion criteria as well as the multivariate analysis adjusting for important confounding factors comprise the main strengths of our study. However, our study has certain limitations. This is a single-center study. Therefore, it is unclear whether our findings apply to other critically ill septic patients. Nonetheless, all patients received a standard of care according to international guidelines [70]. We used healthy subjects and not critically ill patients without sepsis as a control group. Therefore, we did not explore possible alterations of omentin-1 in critical illness of other etiology. Another limitation of our study is that we did not exclude patients with obesity, metabolic syndrome, and cardiovascular diseases, which are associated with decreased serum omentin-1. In order to include only patients with measurements of omentin-1 at the predetermined time points, we excluded patients who were either discharged or died before completing 1 week from enrollment in the study. However, our cases comprise a highly representative sample according to the severity scores and the outcome, which are in line with the mortality rates reported for sepsis and septic shock in the current consensus definitions based on large patients cohorts [44]. Finally, despite appropriate adjustment for confounding factors in the statistical analysis, we cannot exclude residual confounding due to other unmeasured factors.

## 5. Conclusions

In this prospective study, we investigated serum omentin-1 in critically ill patients with sepsis at sepsis onset and 1 week after. We demonstrated that omentin-1 was significantly increased at sepsis onset compared to healthy controls, while it increased further 1 week after. Patients with septic shock at enrollment as well as those who did not survive during the next 28 days presented higher omentin-1 and lower kinetics compared to patients with sepsis and survivors. Finally, we found that higher omentin-1 during the first week of sepsis independently predicted 28-day mortality. Our findings suggest that omentin-1 may be a promising prognostic biomarker of sepsis. There is a need for larger, multicenter, prospective studies to elucidate the pathophysiologic role of omentin-1 in sepsis.

## Figures and Tables

**Table 1:** Demographic and baseline clinical and laboratory data * of patients and controls (*n* = 204).

	Patients (*n* = 102)	Controls (*n* = 102)	*p* Value
Age ^a^, years	64.7 ± 15.6	66.4 ± 10.3	0.35
Gender, male, n (%)	57 (55.9)	57 (55.9)	0.56
BMI ^a^, kg/m^2^	29.9 ± 8.5	28.1 ± 5.01	0.06
Septic shock, n (%)	42 (41.2)	-	
Death within 28 days, n (%)	30 (29.4)	-	
**Severity scores**			
APACHE II ^a^	23 ± 7.2	-	
SOFA ^a^	10 ± 3.3	-	
**Hematologic indices**			
Hemoglobin ^a^, g/L	93 ± 20	147.9 ± 16.3	<0.001
White blood cells ^a^ × 10^9^/L	14.1 ± 8.4	6.97 ± 1.8	<0.001
Platelets ^a^ × 10^9^/L	216.2 ± 118.8	243.8 ± 46.9	0.03
**Coagulation indices**			
Prothrombin time ^a^, s	14.3 ± 4.7	11.9 ± 0.8	<0.001
aPTT ^a^, s	38.9 ± 9.4	34.4 ± 7.3	<0.001
Fibrinogen ^a^, μmol/L	14.49 ± 5.26	9.06 ± 1.3	<0.001
**Metabolic indices**			
Lactate ^b^, mmol/L	2.1 (1–9)	-	
Total Protein ^a^, g/L	50 ± 9	71 ± 4.2	<0.001
Albumin ^a^, g/L	24.6 ± 5.9	46.7 ± 5.6	<0.001
Creatinine ^a^, μmol/L	123.76 ± 70.72	74.26 ± 12.38	0.08
Glucose ^a^, mmol/L	7.97 ± 2.9	5.32 ± 1.16	<0.001
Insulin ^b^, pmol/L	197.9 (88.2–402.8)	73.13 (22.2–430.2)	<0.001
HOMA-IR ^b^	8.9 (3.24–34.5)	2.3 (0.65–23.5)	<0.001
**Inflammatory indices**			
CRP ^b^, mg/L	132 (7–431)	3.4 (0.1–10.9)	<0.001
Procalcitonin ^b^, μg/L	0.9 (0.1–100)	-	-
IL-1β ^b^, ng/L	5.9 (5.9–206)	-	-
IL-6 ^b^ ng/L	27.4 (6–444)	-	-
IL-10 ^b^, ng/L	5 (5–300)	-	-
suPAR ^b^, μg/L	13 (2.1–16.8)	-	-
Omentin-1 ^a^, μg/L	763.3 ± 249.3	451.7 ± 122.3	<0.001

* Values are reported as mean ± SD, and those of highly skewed distributed variables are reported as median (range). Abbreviations: APACHE II, acute physiology and chronic health evaluation score; aPTT, activated partial prothrombin time; BMI, body mass index; CRP, C-reactive protein; HOMA-IR, homeostasis model assessment of insulin resistance; IL, interleukin; SOFA, sequential organ failure assessment score; suPAR, soluble urokinase-type plasminogen activator receptor. ^a^ Mean ± SD, ^b^ median, range.

**Table 2:** Serum omentin-1 and inflammatory biomarkers * of patients with sepsis and septic shock, at baseline and 1 week after enrollment (*n* = 102).

	Upon Enrollment			One Week after Enrollment		
	Sepsis (*n* = 60)	Septic Shock (*n* = 42)	*p* Value	Sepsis (*n* = 60)	Septic Shock (*n* = 42)	*p* Value
CRP ^b^, mg/L	89 (7–218)	174 (36–431)	<0.001	55 (8–282)	101 (13–253)	0.01
Procalcitonin ^b^, μg/L	0.7 (0.09–47.7)	4.8 (0.14–100)	0.002	0.5 (0.06–15)	1.4 (0.14–83)	0.001
IL-1β ^b^, ng/L	5.9 (5.9–207)	8.8 (5.9–44.8)	0.18	17 (5.9–499)	8.8 (5.9–45)	0.13
IL-6 ^b^, ng/L	16.5 (6–385)	74.4 (10–444)	0.001	25 (4.6–419)	20.5 (6–487)	0.34
IL-10 ^b^, ng/L	5 (5–300)	6.9 (5–87)	0.001	5 (5–300)	5 (5–66)	0.02
suPAR ^b^, μg/L	10.5 (2.2–16.8)	14.1 (4.4–16.8)	0.04	11.3 (2.6–16.8)	12.9 (5.2–16.8)	0.68
Omentin-1 ^a^, μg/L	683.1 ± 223.7	877.9 ± 241.2	<0.001	901.7 ± 196.3	1020.4 ± 224.7	0.007

* Values are reported as mean ± SD, and those of highly skewed distributed variables are reported as median (range). Abbreviations: CRP, C-reactive protein; IL, interleukin; suPAR, soluble urokinase-type plasminogen activator receptor. ^a^ Mean ± SD, ^b^ median, range.

**Table 3:** Receiver operator characteristic curve analysis of omentin-1 and significant inflammatory biomarkers at enrollment to determine the optimum cutoff value for the discrimination of sepsis from septic shock in 102 patients with sepsis.

Biomarkers	AUC (95% CI)	*p* Value	Sensitivity	Specificity	Youden’s Index	Cutoff Value	Positive Predictive Value	Negative Predictive Value
Omentin-1	0.74 (0.64–0.84)	<0.001	74%	75%	0.49	850.3 μg/L	67.4%	80.3%
CRP	0.78 (0.68–0.87)	<0.001	80%	69%	0.49	132 mg/L	64.4%	83.1%
Procalcitonin	0.71 (0.60–0.81)	0.001	60%	82.8%	0.43	4.30 μg/L	70.9%	74.7%
IL-6	0.69 (0.58–0.79)	0.001	70%	62.1%	0.32	24.50 ng/L	56.4%	74.7%
IL-10	0.68 (0.57–0.79)	0.003	60%	82.8%	0.43	5.88 ng/L	70.9%	74.7%
suPAR	0.64 (0.53–0.75)	0.02	75%	58.6%	0.34	11.79 μg/L	55.9%	77%

Abbreviations: AUC, area under the curve; CI, confidence interval; CRP, C-reactive protein; IL, interleukin; suPAR, soluble urokinase-type plasminogen activator receptor.

**Table 4:** Multivariate Cox regression analysis for the independent predictors of mortality (expressed as quartiles) after adjustment for the APACHE II score and statistically significant laboratory biomarkers of sepsis in 102 patients.

	b	SE_b_	Wald	df	*p* Value	HR	95% for C.I.
**Independent predictors at enrollment**							
Omentin-1	0.81	0.32	6.59	1	0.01 *	2.26	1.21–4.19
CRP	0.29	0.19	2.51	1	0.11	1.35	0.93–1.95
IL-6	−0.07	0.19	0.13	1	0.71	0.93	0.64–1.35
APACHE II	0.38	0.18	4.49	1	**0.03**	1.46	1.03–2.08
**Independent predictors 1 week after enrollment**							
Omentin-1	0.76	0.21	13.59	1	**<0.001**	2.15	1.43–3.22
CRP	−0.11	1.86	0.34	1	0.56	0.89	0.62–1.29
IL-6	0.68	0.21	10.13	1	**0.001**	1.98	1.30–3.01
APACHE II	0.79	0.23	11.86	1	**<0.001**	2.22	1.41–3.49

Abbreviations: APACHE, acute physiology and chronic health evaluation score; CI, confidence interval; CRP, C-reactive protein; df, degrees of freedom; HR, hazard ratio; IL, interleukin; SE_b_, standard error of b. * Significant results are highlighted in bold.

**Table 5:** Spearman correlation coefficients of serum omentin-1 with laboratory biomarkers in septic patients at enrollment and 1 week after (*n* = 102).

	At Enrollment		One Week after Enrollment	
	r	*p*	r	*p*
**Hematologic biomarkers**				
Hemoglobin	−0.04	0.67	−0.04	0.7
White blood cells	0.28 *	**0.004**	**0.23**	**0.02**
Neutrophils	0.1	0.3	**0.2**	**0.04**
Platelets	−0.03	0.76	−0.1	0.29
**Coagulation biomarkers**				
Prothrombin time	**0.4**	**<0.001**	**0.31**	**0.002**
aPTT	**0.27**	**0.006**	0.18	0.08
Fibrinogen	**0.24**	**0.02**	0.14	0.18
**Metabolic biomarkers**				
Lactate	**0.19**	**0.05**	0.16	0.11
Total protein	−0.16	0.09	−0.15	0.13
Albumin	−0.13	0.2	−0.14	0.16
Creatinine	**0.24**	**0.01**	0.16	0.11
Glucose	0.11	0.33	-	-
Insulin	0.28	0.15	-	-
HOMA-IR	**0.3**	**0.002**	-	-
BMI	−0.07	0.48	-	-
**Inflammatory biomarkers**				
CRP	**0.41**	**<0.001**	0.15	0.13
Procalcitonin	0.08	0.41	−0.02	0.85
IL-1β	0.11	0.26	−0.17	0.09
IL-6	0.1	0.31	−0.02	0.84
IL-10	0.19	0.05	−0.07	0.49
suPAR	0.09	0.34	0.05	0.63

Abbreviations: aPTT, activated partial thromboplastin time; BMI, body mass index; CRP, C-reactive protein; HOMA-IR, homeostasis model assessment of insulin resistance; IL, interleukin; suPAR, soluble urokinase-type plasminogen activator receptor. * Significant correlations are highlighted in bold.

## Data Availability

Data to support the findings of this study are available upon reasonable request.
